# Characterization of GUA-1, a chromosomally encoded ESBL from *Pseudomonas guariconensis-like*

**DOI:** 10.1128/aac.00126-26

**Published:** 2026-06-30

**Authors:** Saoussen Oueslati, Nadia Jaidane, Reva Nermont, Delphine Girlich, Nahed Al Laham, Lamia Tilouche, Wejdene Mansour, Marisa Haenni, Bogdan I. Iorga, Thierry Naas

**Affiliations:** 1 Team 'Resist', UMR1184 IDMIT (INSERM, Université Paris-Saclay, CEA), Faculty of Medicine, Le Kremlin-Bicêtre, France; 2 Department of Bacteriology-Hygiene, Bicêtre Hospital, APHP Paris-Saclay 537860, Le Kremlin-Bicêtre, France; 3 Laboratory of Metabolic Biophysics and Applied Pharmacology (LR12ES02), Department of Biophysics, Faculty of Medicine Ibn El Jazzar of Sousse, University of Sousse 37961, Sousse, Tunisia; 4 Clinical Microbiology Laboratory, University Hospital of Sahloul, Sousse, Tunisia; 5 Laboratory Medicine Department, Faculty of Applied Medical Sciences, Al Azhar University-Gaza 117301, Gaza, Palestine; 6 ANSES-Université de Lyon, Unité Antibiorésistance et Virulence Bactériennes, Lyon, France; 7 Université Paris-Saclay, CNRS, Institut de Chimie des Substances Naturelles (ICSN) 27048, Gif-sur-Yvette, France; 8 Health and Therapeutic Innovation object (Healthi), Université Paris-Saclay, Orsay, France; 9 French National Reference Center for Antibiotic Resistance: Carbapenemase-producing Enterobacterales, Le Kremlin-Bicêtre, France; 10 SEPSIS Comprehensive Center–IHU SEPSIS, Le Kremlin-Bicêtre, France; Universita degli Studi di Roma La Sapienza, Rome, Italy

**Keywords:** extended spectrum beta-lactamase, class A, cephalosporinase, *Pseudomonas *sp

## Abstract

Putative novel β-lactamase-encoding genes are increasingly identified in whole-genome sequencing (WGS) data, often without phenotypic or biochemical characterization. Here, we characterized the chromosome-encoded GUA-1 Ambler class A β-lactamase from the clinical isolate *Pseudomonas guariconensis-like* 65411. Among the 40 *P. guariconensis* genomes available in the GenBank database, only 8 carried a *bla*_GUA-like_ gene, whereas the others encoded an AmpC β-lactamase, suggesting the existence of two distinct subspecies. Notably, these eight isolates, despite originating from diverse geographical regions, all harbored the *bla*_GUA-like_ gene inserted at the same chromosomal locus. Heterologous expression in *Pseudomonas aeruginosa* and *Escherichia coli* revealed a clavulanic-acid-inhibited cefotaximase-type ESBL. GUA-1 shared 72% amino acid sequence identity with putative Ambler class A β-lactamases from *Pseudomonas fulva*, *Pseudomonas mosselii*, and *Pseudomonas soli*, and 57% with LUT-1, a class A cephalosporinase from *Pseudomonas luteola*. Kinetic analyses showed that GUA-1 hydrolyzed cefotaxime but not ceftazidime, similarly to LUT-1 and CTX-M-1-type ESBLs. IC_50_ measurements indicated strong inhibition by clavulanic acid, tazobactam, and avibactam, but not by vaborbactam. Molecular modeling supported these findings, showing favorable binding of cefotaxime in the active site, whereas steric hindrance from Trp274 and electrostatic repulsion from Asp240 likely prevent ceftazidime binding. The *bla*_GUA-1_ gene was chromosomally located, with no obvious promoter identified in the immediate upstream region. RT-PCR experiments revealed expression levels comparable to other β-lactamase genes present (*bla*_DHA_, *bla*_CMY_, *bla*_OXA-1_, and *bla*_OXA-204_) and to the upstream-located *Na+/H* antiporter gene. 5′ RACE experiments suggested that the *bla*_GUA-1_ gene is co-transcribed along with the *Na+/H* gene. Overall, this study highlights the diversity of β-lactamases within *Pseudomonas* species.

## INTRODUCTION

The number of β-lactamases described in the β-lactamase database (BLDB) is constantly increasing, with more than 12,741 enzymes (http://www.bldb.eu, last accessed January 2026) (1). This increase is mainly driven by the diversification of clinically relevant enzymes, best exemplified by CTX-M-type ESBL and KPC-type carbapenemases (with more than 283 and 284 variants, respectively), as well as by whole-genome sequencing (WGS), which identifies novel chromosomally encoded β-lactamases but generally provides no information on their hydrolysis profiles nor on their contribution to the overall resistance phenotype, even though some of these enzymes are considered surrogate markers of the bacterial species in which they were identified.

*Pseudomonas guariconensis* was initially isolated from rhizospheric soils in 2013 in Guárico, Venezuela (2), and was subsequently recovered from environmental samples collected in the Brazilian Amazon Basin, where it was associated with a novel acquired MBL, named BIM-1 (3). Since then, it has been sporadically described in human infections, including endocarditis, necrotizing fasciitis, bacteriuria, and bacteremia, in some cases exhibiting a highly drug-resistant phenotype associated with the presence of VIM-2, VIM-5, or OXA-204 carbapenemases (4–8). The WGS of *P. guariconensis-like* 65411 clinical isolate revealed a resistome comprising several acquired β-lactam resistance genes (*bla*_CMY-16_, *bla*_DHA-1_, *bla*_OXA-1_, *bla*_OXA-10_, and *bla*_OXA-204_), but no chromosome-encoded *bla*_AmpC_ gene. Instead, an ORF 100% identical to an uncharacterized putative β-lactamase gene found in the genome of an unpublished *P. guariconensis-like* isolate of animal origin from the Czech Republic (UQM99659.1) (5). This ORF contained all the conserved serine active site motifs suggesting a functional class A β-lactamase named GUA-1 (5, 9). Crude protein extracts of the cloned *bla*_GUA-1_ in *E. coli* revealed strong nitrocefin hydrolytic activity and yielded a positive β-Lacta test (Bio-Rad, Marne-la-Coquette, France), suggesting expanded-spectrum cephalosporin (ESC) hydrolytic activity (5).

Here, the *bla*_GUA-1_ gene was expressed in *E. coli* Top10 and *P. aeruginosa* KG2505 (ΔAmpC) to study the conferred β-lactam susceptibility profile, and the steady-state kinetic parameters of purified GUA-1 were determined. In addition, *bla*_GUA-1_ expression in *P. guariconensis-like* 65411 was investigated using RT-PCR and 5′ RACE experiments.

## RESULTS AND DISCUSSION

### Sequence analysis and phylogeny of GUA-1 β-lactamase

**Fig 1:**
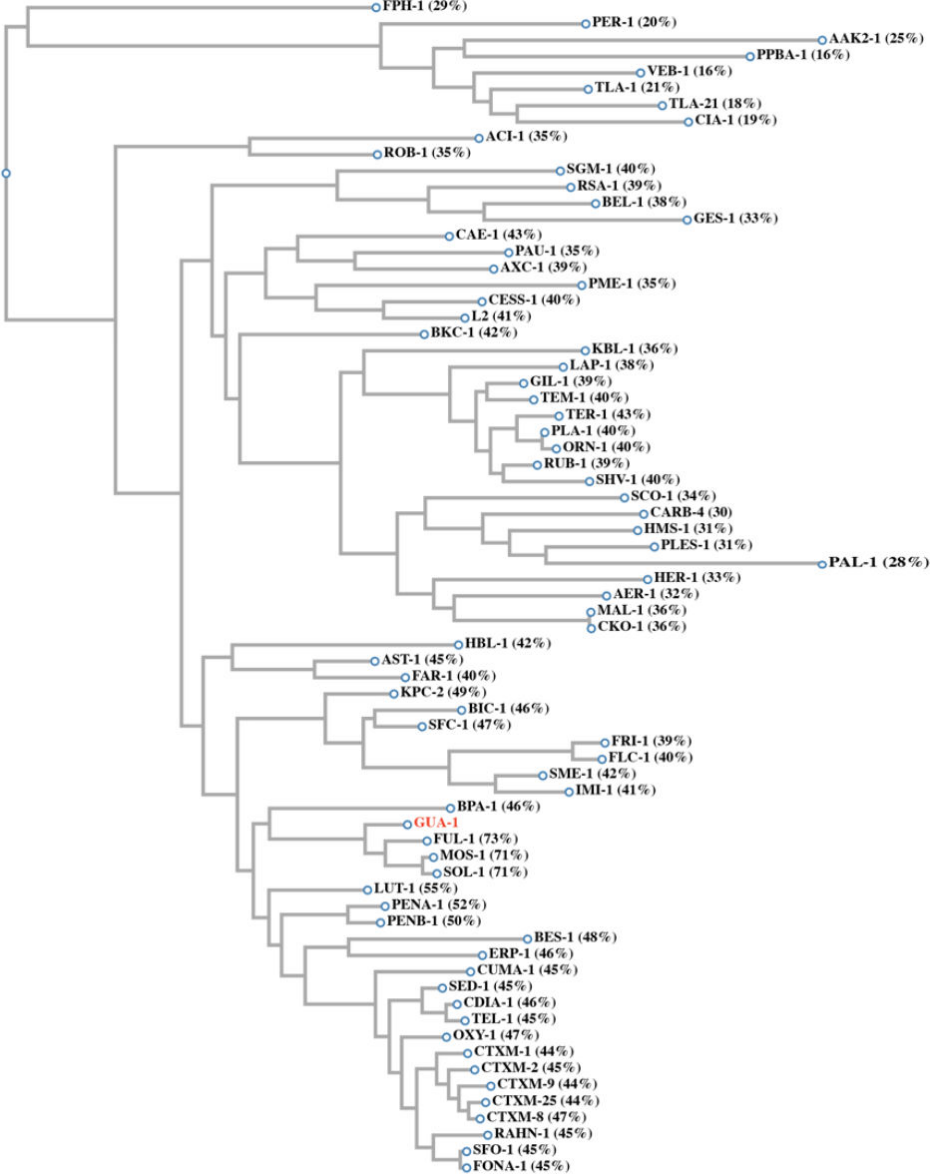
Phylogeny of the full-length amino acid sequences of GUA-1 and 71 other class A β-lactamases from the BLDB (http://bldb.eu/) constructed using ClustalW aligned amino acid sequences (https://www.genome.jp/tools-bin/clustalw) and PhyML v20160115 (11). Branch lengths are drawn to scale and are proportional to the number of amino acid changes. The distance along the vertical axis has no significance. The amino acid identity of each β-lactamase with that of GUA-1 from *P. guariconensis* is indicated in parentheses.

A phylogenetic study was carried out to assess the relationship between GUA-1 with representative members of the various branches of naturally occurring and acquired class A β-lactamases present in the BLDB (Fig. 1) (1). The predicted GUA-1 protein of 291 amino acids (AA) showed similarities to several other chromosome-encoded putative class A β-lactamases identified in gram-negative bacteria, especially with 72% *Pseudomonas fulva* (FUL-1; MBF8778391.1), 71% *Pseudomonas mosselii* (MOS-1; WP_345894069.1), and *Pseudomonas soli* (SOL-1; UXZ44560.1), and 57% with the characterized ESC-hydrolyzing LUT-1 from *Pseudomonas luteola* (10). The GUA-1 β-lactamase also shared low identities with chromosomal-class A cephalosporinase (PenA from *Burkholderia cepacia* complex [52%], OXY-5 from *Klebsiella oxytoca* [47%], SED-1 from *Citrobacter sedlakii* [45%], FONA-1 from *Serratia fonticola* [45%], CTX-M family from *Kluyvera* spp. [44%]), but also with class A carbapenemases such as KPC-1 from *Klebsiella pneumoniae* and SFC-1 from *S. fonticola* (Fig. 1).

**Fig 2:**
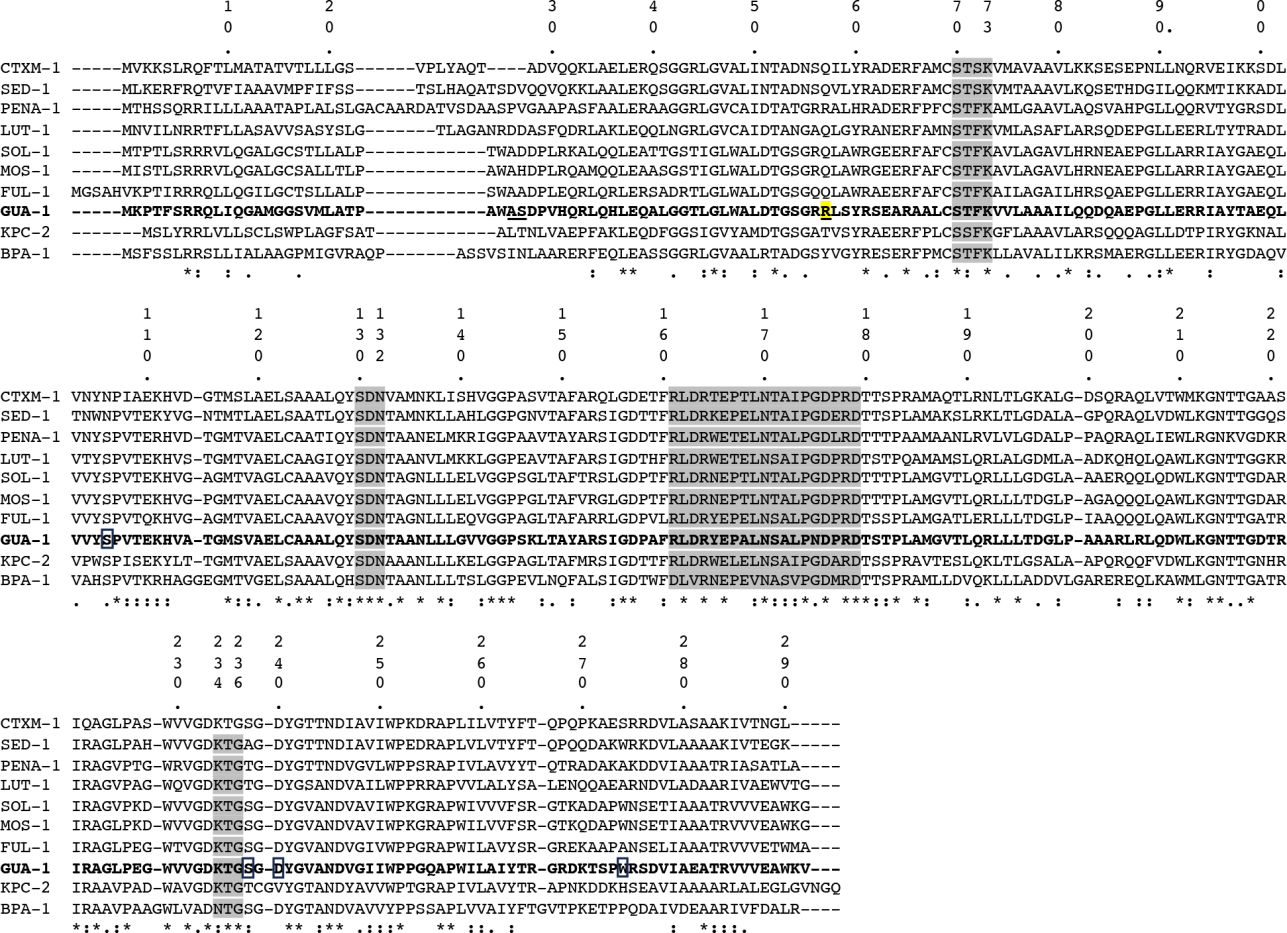
Amino acid sequence alignment of GUA-1 from *P. guariconensis*-like 65411, FUL-1 from *P. fulva* (MBF8778391.1), SOL-1 from *P. soli* (UXZ44560.1), MOS-1 from *P. mosselii* (WP_186595297.1), LUT-1 from *P. luteola* (AAU10324.1), BPA-1 from *Paraburkholderia phymatum* STM815 (NC_010622), PENA-1 from *Burkholderia multivorans* ATCC 17616 (U85041), CTX-M-1 (DQ915955), and SED-1 from *Citrobacter sedlakii* (AF321608). Conserved motifs among class A beta-lactamases are highlighted in gray. Ser106, Ser237, and Arg276 are boxed. The sequence of GUA-1 has been bolded.

Class A conserved serine active site motifs (S_70_XXK), the hydrolytic water-binding site (S_130_DN), the omega loop, and the catalytic triad component (K_234_TG) (10), along with particular residues such as Ser106, Ser237, and Arg275 (Fig. 2), identified as important positions for the action against oxyiminocephalosporins in other ESBLs (TEMs and CTX-Ms), were found in GUA-1 (9). In addition, Asp240 that has been shown to establish hydrogen bonds with the amide and aminothiazole groups of the acyl-amide-cefotaxime chain, thus allowing tight binding of cefotaxime in the binding site, and that is preventing binding of ceftazidime through repulsion due to the carboxylic acid group on oxyimino substituents of ceftazidime, is present in GUA-1 (12–14). Using SignalP5.0, a leader peptide cleavage site located between AA A27-S28 was determined, resulting in a 264 AA long mature GUA-1 protein.

*Bla*_GUA_-type β-lactamase genes have been identified in the genomes of eight additional *P. guariconensis* isolates from an environmental source in India (*n* = 1), from human origin in France (*n* = 1), and from animal origin in Czech Republic (*n* = 6) (Table S2). Alignment of the genes and the deduced proteins revealed that 6/7 were identical to GUA-1, while one isolate expressed a R57Q variant, and was named GUA-2 (Fig. S1 and S2). Among the 40 *P. guariconensis* genomes available in the GenBank database, 22 harbored a distinct class A β-lactamase only distantly related to GUA-1 (≈35% amino acid identity) and/or a chromosomally encoded AmpC enzyme, both of which are absent in GUA-1–producing strains. This distribution suggests the existence of a distinct lineage within *P. guariconensis*, potentially corresponding to a subspecies. Accordingly, we designated our isolate as *P. guariconensis*-like, supporting the hypothesis that strain 65411 may represent a subspecies or even a novel species, as previously proposed by Jaidane et al. (5).

### Antimicrobial susceptibility conferred by GUA-1

*E. coli* Top10 harboring plasmid pTOPO-GUA-1 and *P. aeruginosa* KG2505 harboring pUCP24-GUA-1 were used for disk diffusion antibiograms (data not shown) and for MIC determination using 12 β-lactam antibiotics (Table 1).

**TABLE 1:** MICs of *bla*_GUA-1_ expressed in *E. coli* TOP10 and in *P. aeruginosa* KG2505, in comparison with those of *bla*_LUT-1_ β-lactamase expressed in *E. coli* DH10B, derived from Doublet et al. (10)

Beta-lactams	MIC (µg/mL)^*a*^					
	*E. coli* TOP10 pTOPO-GUA-1	*E. coli* TOP10 pTOPO	*E. coli* DH10B pBK-L3 (LUT-1)*^b^*	*E. coli* DH10B*^b^*	*P. aeruginosa* KG2505 pUCP24 GUA-1	*P. aeruginosa* KG2505 pUCP24
Amoxicillin	12	2	128	4	48	0.19
Amoxicillin + CLA^*c*^	4	2	4	4	1.5	0.094
Ticarcillin	64	4	256	2	128	1
Ticarcillin + CLA^*c*^	8	4	16	2	16	1
Piperacillin^*c*^	4	2	NT^*d*^	NT	2	<0.25
Piperacillin + TZP^*e*^	2	2	NT	NT	<0.25	<0.25
Cephalothin	64	8	32	4	128	16
Cefoxitin	8	4	4	4	1	0.19
Cefotaxime	0.094	0.06	1	<0.06	4	0.125
Ceftazidime	0.38	0.12	1	0.125	0.75	0.75
Ceftazidime + AVI^*f*^	0.25	0.25	NT	NT	0.5	0.5
Cefepime	0.032	0.023	0.5	0.06	1.5	0.25
Aztreonam	0.125	0.047	2	<0.06	0.25	0.094
Imipenem	0.19	0.19	0.5	0.5	0.38	0.25

^*a*^ MICs were determined using E-Tests (bioMérieux) except for underlined antibiotics that were determined using broth microdilution.

^*b*^ Data were from Doublet et al. (10).

^*c*^ CLA, clavulanic acid; 2 mg/L.

^*d*^ NT, not tested by Doublet et al. (10).

^*e*^ TZP, Tazobactam at a fixed concentration of 4 µg/mL.

^*f*^ AVI, avibactam; 4 mg/L.

On routine antibiogram, overall decreased inhibition diameters were observed as compared to parental bacteria lacking the *bla*_GUA-1_ gene (data not shown). Determination of MIC values confirmed the disk diffusion results. In *E. coli*, GUA-1 conferred reduced susceptibility to most tested β-lactams, except for imipenem; however, MIC values remained low, likely due to expression problems in that species (Table 1). The MIC values obtained for GUA-1 were lower than those previously reported for LUT-1 (10), although the overall susceptibility trends were similar in both strains. These differences may be explained by AA sequence differences between LUT-1 and GUA-1, or by differences in the *E. coli* host strain, the size of the cloned DNA fragment, and the cloning plasmid used. In *P. aeruginosa* lacking its native AmpC enzyme, GUA-1 conferred resistance to penicillins and to first-generation cephalosporins, as well as reduced susceptibility to ESCs (including cefotaxime, cefepime, and aztreonam with MICs of 4 µg/mL, 1.5 µg/mL, and 0.25 µg/mL, respectively). The addition of clavulanic acid restored susceptibility to penicillins, consistent with an ESBL phenotype.

### Biochemical properties of GUA-1

Kinetic parameters indicated that the GUA-1 enzyme had a broad substrate profile, including penicillins and cephalosporins, such as cephalothin, and cefotaxime in particular (Table 2). The catalytic efficiency (*k*_cat_*/K*_m_) of benzylpenicillin was the highest and comparable to that of cephalothin, while that of amoxicillin was comparable to that of cefotaxime (Table 2). As revealed by low *K*_m_s, the affinity for penicillins was higher than for cephalosporins, but the *k*_cat_ was higher for cephalosporins compared with penicillins. For imipenem and ceftazidime, no hydrolysis could be detected. Similar to CTX-Ms, GUA-1 displayed stronger hydrolytic activity against cefotaxime than against ceftazidime or aztreonam (*k*_cat_, 67 s^−1^ versus no hydrolysis or 5.1 s^−1^, respectively).

**TABLE 2:** Steady-state kinetic parameters for GUA-1 in comparison with those of LUT-1 β-lactamase, derived from Doublet et al. (10)

	GUA-1			LUT-1^*a*^		
	*K*_m_ (μM)	*k*_cat_ (s^−1^)	*k*_cat_/*K*_m_ (mM^−1^/s^−1^)	*K*_m_ (μM)	*k*_cat_ (s^−1^)	*k*_cat_/*K*_m_ (mM^−1^/s^−1^)
PeniG	6.4 ± 0.6	41.5 ± 3.9	6,514 ± 86	8	31	3,875
Amoxicillin	7.0 ± 0.6	18.3 ± 2.7	2,602 ± 357	20	21	1,050
Ticarcillin^*b*^	27.0 ± 2.0	2.8 ± 0.7	103.6 ± 20	14	5	357
Cephalotin	36.7 ± 8.3	224.5 ± 24.3	6,227 ± 691	21	543	25,857
Cefoxitin	NH^*c*^	NH	NH	ND^*d*^	ND	ND
Ceftazidime	NH	NH	NH	35	1.3	37
Cefotaxime	57.3 ± 4.2	67.0 ± 2.0	1,171 ± 53	86	452	5,256
Cefepime	218.0 ± 13.5	11.1 ± 1.8	50.9 ± 7.5	ND	ND	ND
Imipenem	NH	NH	NH	ND	ND	ND
Aztreonam	286.7 ± 12.7	5.1 ± 0.1	17.9 ± 0.9	40	1.5	37

^*a*^ Data were from Doublet et al. (10).

^*b*^ Weak hydrolysis requiring undiluted enzyme preparation (concentration of 4 µg/mL), thus could only be done once.

^*c*^ NH, no detectable hydrolysis with 4 µg/mL of enzyme and 500 µM of ceftazidime.

^*d*^ ND, not done by Doublet et al. (10).

IC_50_ determinations revealed that GUA-1 was inhibited by low concentrations of clavulanic acid, tazobactam, and avibactam (IC_50_, 133 nM; 73.5 nM, and 54 nM, respectively) but only moderately by vaborbactam (IC_50_ 4.7 µM). Given its susceptibility to clavulanate and its ability to hydrolyze ESCs, GUA-1 can be classified as an ESBL and as a member of the 2e group in the functional classification of β-lactamases (15). Vaborbactam, which is a cyclic boronic acid, mimics the tetrahedral transition state of β-lactam hydrolysis and forms a reversible covalent boronate complex with Ser70. However, its binding relies more on precise active-site geometry and fewer stabilizing interactions than avibactam. As a result, it shows very high potency against KPC enzymes but a narrower spectrum overall within class A, with weaker activity against some ESBLs or enzymes with structural variations in the binding pocket (16).

### Structural feature of GUA-1

**Fig 3:**
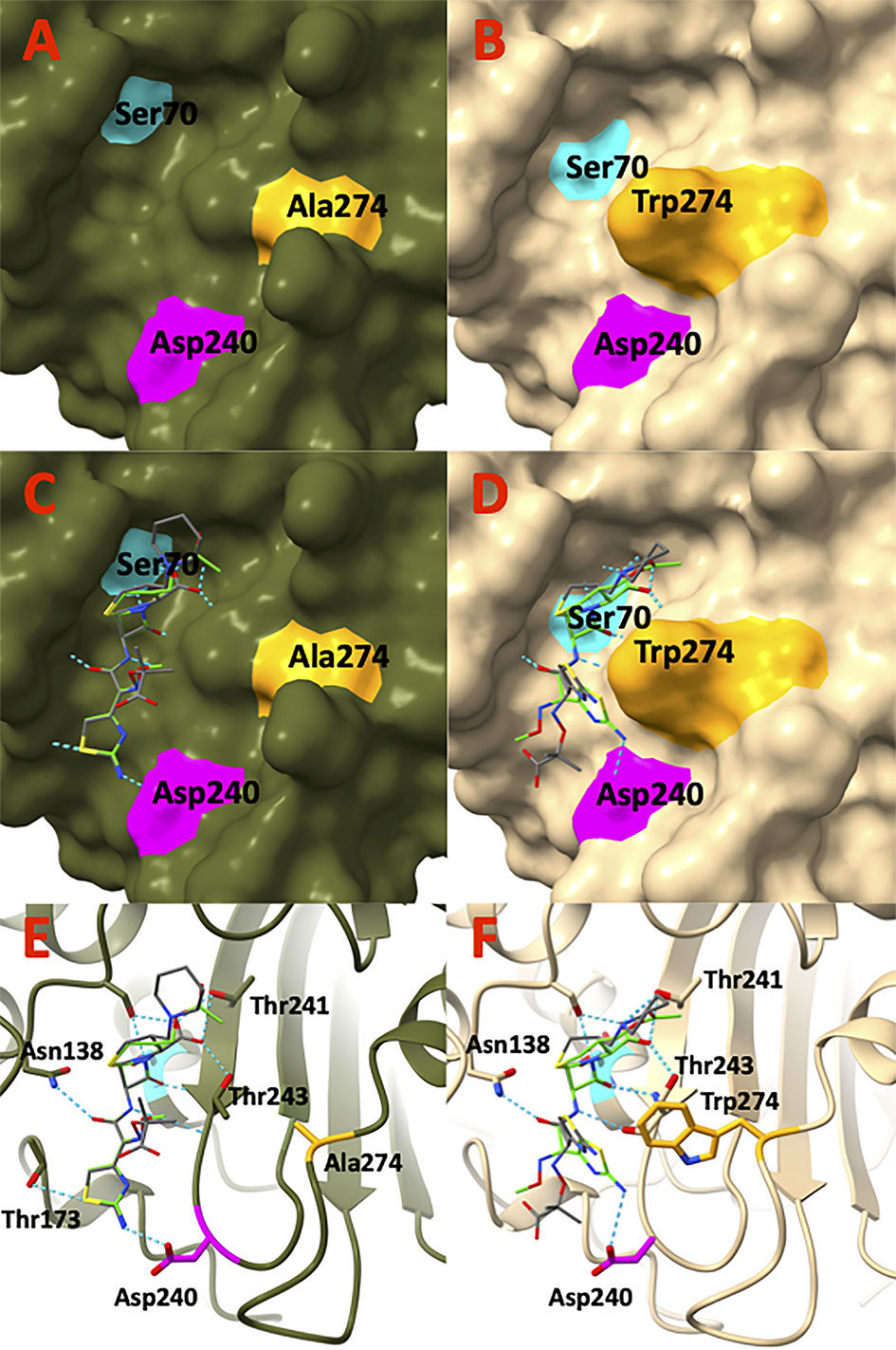
Representation of the predicted 3D structures for LUT-1 (**A and C**—olive color) and GUA-1 (**B and D**—cream color) in apo form (**A and B**—surface) or in complex with cefotaxime (green) and ceftazidime (gray) (**C and D**—surface; **E and F**—ribbon). The ligands are shown in stick representation, with hydrogen bonds drawn as dashed lines. The key residues Ser70, Asp240, and Ala/Trp274 are colored in cyan, magenta, and orange, respectively.

The overall fold of GUA-1 is similar to that of other closely related class A β-lactamases. However, compared with LUT-1, the active site of GUA-1 is markedly narrower because residue 274 is bulkier (Trp in GUA-1 versus Ala in LUT-1) (Fig. 3A and B). Under these conditions, LUT-1 can accommodate both cefotaxime and ceftazidime without steric hindrance, in a canonical binding mode that includes a hydrogen bond between the aminothiazole group and the Asp240 side chain (Fig. 3C). In GUA-1, this binding mode is precluded by steric clashes between Trp274 and the oxyimino groups of cefotaxime and ceftazidime, which forces a rotation of the aminothiazole/oxyimino substituents. For cefotaxime, this alternative conformation can still be accommodated in the GUA-1 active site. In contrast, for ceftazidime, an additional electrostatic repulsion between the oxyimino group and Asp240 further disfavors binding, effectively preventing productive complex formation (Fig. 3D) as previously shown for CTX-M enzymes (14). In CTX-M enzymes, Asp240 forms hydrogen bonds with the amide and aminothiazole groups of the acyl-amide cefotaxime side chain, thereby promoting tight binding of cefotaxime within the active site. In contrast, the presence of Lys or Arg at position 240 in TEM and SHV ESBLs enhances activity against ceftazidime by enabling electrostatic interactions with the carboxylate group of its oxyimino substituent (17). Conversely, CTX-M variants carrying an Asp240Gly substitution (e.g., CTX-M-15, CTX-M-16, and CTX-M-27) lack this electrostatic interaction but may facilitate accommodation of the bulky oxyimino-ceftazidime side chain.

This structural analysis is fully consistent with the kinetic parameters reported in Table 2 and with those reported for SED-1, a class A cefotaximase that hydrolyzes penicillins and some expanded-spectrum cephalosporins (notably cefotaxime), but shows little to no activity against ceftazidime (18). In class A β-lactamases, position 274 plays an important structural and functional role in shaping the active site, particularly its distal region, often referred to as the “R2 binding pocket,” which accommodates bulky cephalosporin side chains. In the case of Trp274, as shown in SED-1, its indole side chain partially protrudes into the substrate-binding cavity, which restricts access or proper positioning of β-lactams that carry bulky oxyimino side chains, such as ceftazidime (18). This steric hindrance is thought to limit hydrolysis of such substrates, even when other catalytic features are compatible. Thus, it is likely that in GUA-1, Trp274 contributes to a more constrained active site architecture, influencing substrate specificity.

Interestingly, cefepime, which is also an oxyimino cephalosporin, is, unlike ceftazidime, hydrolyzed by GUA-1. A possible explanation is that ceftazidime’s oxyimino side chain is larger and more flexible, whereas that of cefepime is smaller and more compact, resulting in less steric hindrance and thus explaining their distinct hydrolysis by GUA-1

### Genetic environment of *bla*_GUA-1_ gene

**Fig 4:**
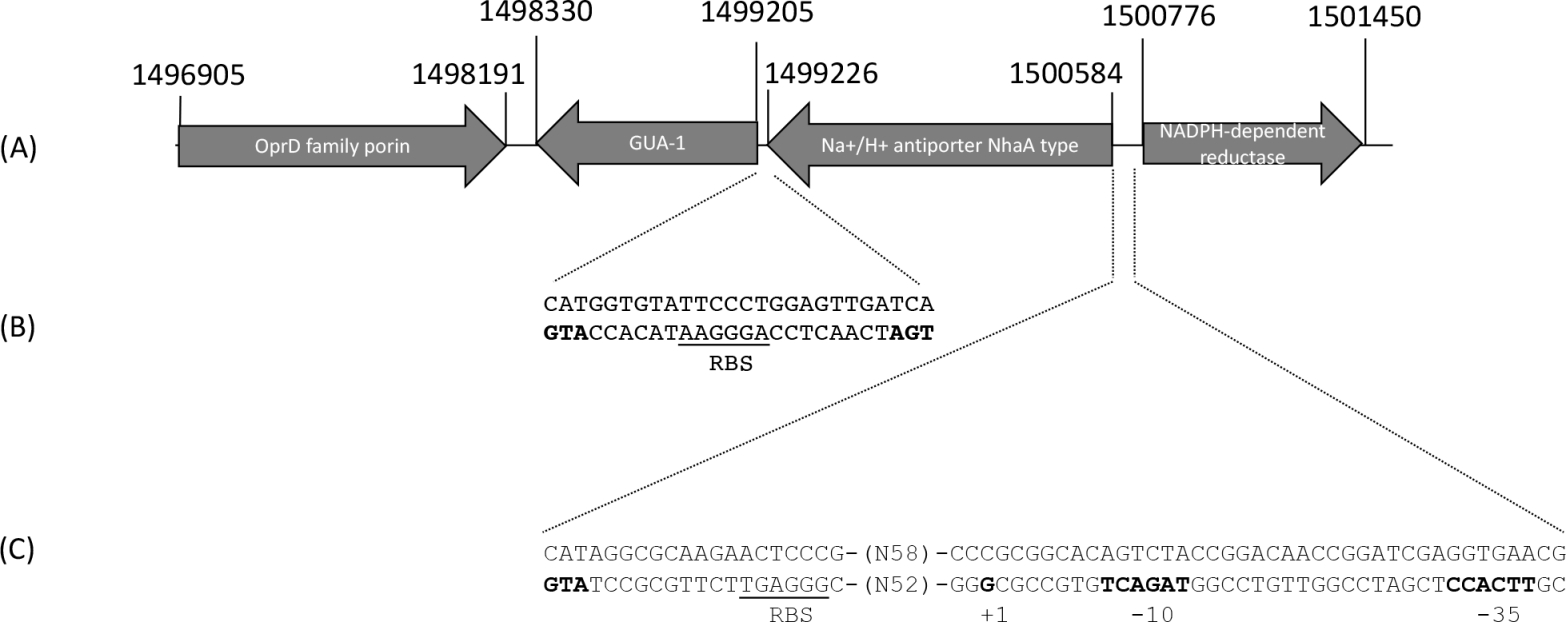
Genetic environment of the *bla*_GUA-1_ gene. (**A**) Genes and their orientation are indicated by arrows. (**B**) Intercistronic region between the *bla*_GUA-1_ and *Na+/H* + antiporter genes. Start (ATG) and stop (TGA) codons are bolded, and ribosomal-binding site (RBS) underlined; and (**C**) 5′ RACE determined promoter of the *Na+/H* + antiporter gene. *Bla*_GUA-1_ start (ATG), +1 determined by 5′ RACE and deduced −10 and −35 boxes are bolded. RBS is underlined.

**Fig 5:**
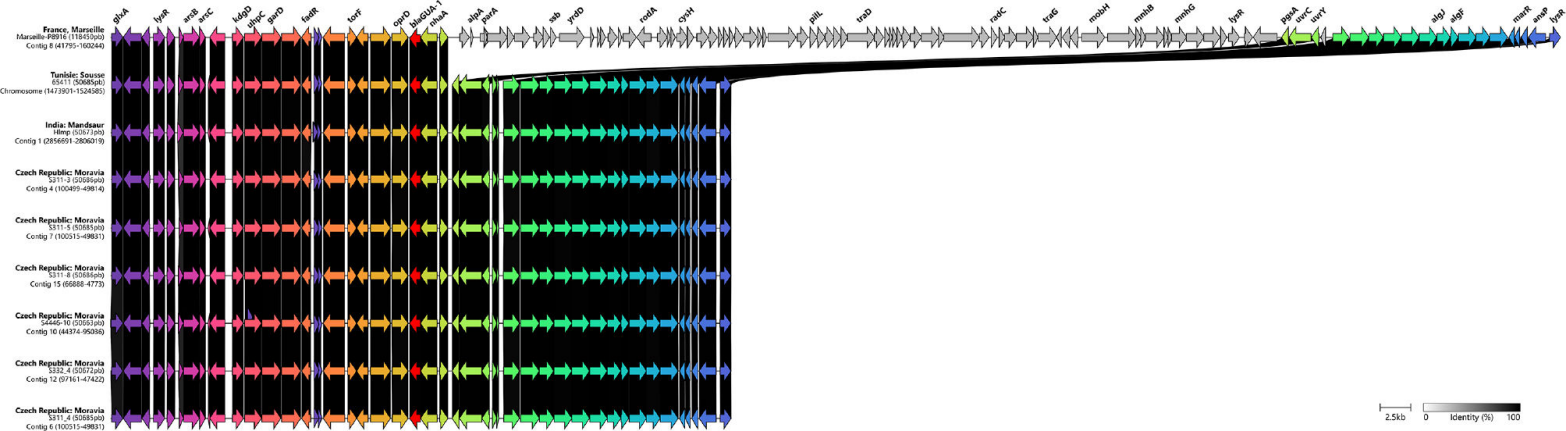
Comparison of the genetic environment of the *bla*_GUA-1_ gene in different *P. guariconensis*-like 65411 isolates, using the Clinker web-based tool (https://cagecat.bioinformatics.nl/tools/clinker). The country, city, and the name of the isolates are indicated, along with the size of the fragment and its position within a contig or the chromosome or the contig. The nucleotide sequence identity between each coding region is represented with a color gradient ranging from white (0% identity) to black (100% identity). Genes of interest are highlighted, and *bla*_GUA-1_ is indicated in red.

*Bla*_GUA-1_ gene is chromosomally located in *P. guariconensis*-like 65411, and no putative Lys-R-type regulator was found directly upstream of it, suggesting a constitutive expression. The upstream region contained a *Na+/H* + antiporter NhaA-type gene, and the downstream region a 1286-bp ORF encoding an OprD family porin (Fig. 4A). No obvious promoter could be predicted using the online tool SAPPHIRE (SAPPHIRE.CNN.pseudomonas; https://sapphire.biw.kuleuven.be/index.php?) upstream of the *bla*_GUA-1_ gene. A putative ribosomal binding site (RBS) was identified five nucleotides upstream of the ATG within the 25-bp intergenic region (Fig. 4B). A putative promoter sequence was predicted upstream of the *Na+/H* + antiporter gene. The expression of the *bla*_GUA-1_ gene in *P. guariconensis*-like 65411 could thus be mediated by the promoter of the *Na+/H* + antiporter gene, through a polycistronic mRNA. Finally, no target site duplication, nor mobile elements were found associated with the *bla*_GUA-1_ gene. Analysis of the immediate genetic environment of a *bla*_GUA-like_ gene in GUA-1-producing *P. guariconensis*-like 65411 isolates revealed >99.0% nucleotide identity upstream and downstream of the β-lactamase gene (Fig. 5).

### Expression of *bla*_GUA-1_ gene

To study whether the *bla*_GUA-1_ gene is expressed or corresponds to a likely cryptic ORF in *P. guariconensis*-like 65411, the transcription level of the *bla*_GUA-1_ gene was determined and compared to those of the other β-lactamases expressed in *P. guariconensis*-like 65411 (Table 3). Results were normalized against Na+/H + antiporter gene. The expression level of *bla*_GUA-1_ was comparable to that of the other β-lactamases and *Na+/H* + gene. Only *bla*_OXA-10_ displayed a 43-fold higher expression as compared to the *bla*_GUA-1_ gene, likely because of the strong integron-borne Pc promoter that drives gene cassette expression in class 1 integrons (19).

**TABLE 3:** Expression of β-lactamase genes as measured by quantitative qRT-PCR, present in *P. guariconensis*-like 65411

Gene	Average Ct (SD)^*a*^	Ratio Ct/Ct NA/H	ΔCt (Ct − Ct NA/H)	Relative expression (2^−ΔCt^)
*bla*_GUA-1_	22.67 (0.6)	1.07	1.42	0.37
*bla*_DHA-1_	22.43 (1.3)	1.06	1.18	0.44
*bla*_CMY-16_	26.64 (0.6)	1.25	5.39	0.02
*bla*_OXA-1_	20.61 (1.0)	0.97	−0.64	1.56
*bla*_OXA-204_	20.15 (1.0)	0.95	−1.10	2.14
*bla*_OXA-10_	17.24 (0.5)	0.81	−4.01	16.11
*NA+/H* + antiporter	21.25 (0.6)	1.0	0.00	1.00

^*a*^ The results shown are the means of three separate experiments. Standard deviation (SD) is indicated into brackets.

### Mapping of *bla*_GUA-1_ transcription start site

Since no obvious putative *bla*_GUA-1_ promoter could be identified *in silico*, 5′ RACE experiments were undertaken to identify the transcription start site (TSS) of the *bla*_GUA-1_ gene. Despite repeated attempts, no PCR product could be amplified, suggesting that transcription initiation is located further away from the beginning of the *bla*_GUA-1_ coding sequence. Using primers located within the beginning of *Na+/H+*, a + 1 TSS (“G”) was mapped 78-bp upstream of the *Na+/H* + antiporter coding sequence with downstream located –35 promoter sequence (TTCACC) separated by 17 bp from a –10 promoter sequence (TAGACT) (Fig. 4C). Thus, it is very likely that *bla*_GUA-1_ expression is mediated by the promoter of the *Na+/H* + antiporter gene; moreover, as no transcription stop site is present at the end of the *Na+/H* + antiporter gene, it is very likely that transcription read through occurs resulting in a polycistronic mRNA made of *Na+/H* + and *bla*_GUA-1_ coding sequences. This result is in line with *in silico* promoter prediction results and with the fact that the intergenic region of 25 bp is large enough to contain a putative RBS sequence, but too small to contain a promoter sequence.

### Conclusions

The number of β-lactamases listed in the BLDB has increased dramatically since 2016, mainly due to the spread and diversification of clinically relevant, plasmid-encoded enzymes such as CTX-M and KPC (1). This growth also reflects the identification of numerous putative chromosomally encoded β-lactamases from whole-genome sequencing data, although these enzymes are rarely functionally characterized in terms of expression, activity, or contribution to antibiotic resistance. Their characterization is nonetheless essential, both for anticipating potential mobilization and for improving future *in silico* susceptibility prediction models.

Whole-genome sequencing of the OXA-204–producing *P. guariconensis*-like 65411 clinical isolate revealed multiple acquired β-lactam resistance genes, as well as a putative chromosomally encoded class A β-lactamase, *bla*_GUA-1_, identical to unpublished genes previously identified in an animal-derived *P. guariconensis-like* isolate from the Czech Republic, *Pseudomonas* sp isolates from France and India (5). However, out of 40 genomes submitted as *P. guariconensis* only 8, in addition to isolate *P. guariconensis-like* 65411 harbored a *bla*_GUA-like_ gene, suggesting that it could be a surrogate marker of a subspecies of *P. guariconensis,* as other closely related and chromosome-encoded class A β-lactamases identified in other *Pseudomonas* spp., such as FUL-1 in *P. fulva*, MOS-1 in *P. mosselii*, SOL-1 in *P. soli*, and LUT-1 in *P. luteola* are considered species-specific (10). Biochemical and inhibition data demonstrated that GUA-1 displays properties consistent with class A ESBLs, placing it within group 2e of the Bush and Jacoby classification (15). Molecular modeling was able to explain the experimentally observed biochemical differences between LUT-1 and GUA-1, by showing that cefotaxime can bind the GUA-1 active site, whereas ceftazidime binding is hindered by steric clashes with Trp274 and electrostatic repulsion from Asp240. We were able to show that the *bla*_GUA-1_ gene is expressed in *P. guariconensis*-like 65411 at a similar level as the other acquired β-lactamase genes. Interestingly, the *bla*_GUA-1_ gene lacks any promoter sequence, and expression is likely mediated by the promoter of the *Na+/H* + gene, located upstream of *bla*_GUA-1_ and in the same orientation.

Finally, our work highlights the diversity of naturally occurring β-lactamases in *Pseudomonas* spp. and contributes to a better understanding of chromosomally and naturally encoded β-lactamases in that genus.

## MATERIALS AND METHODS

### Bacterial strains

*P. guariconensis-like* 65411 clinical isolate was used for RT-PCR experiments, 5′ RACE, and amplification of the *bla*_GUA-1_ gene (5). *Escherichia coli* TOP10 (Invitrogen, Saint-Aubin, France) and *E. coli* BL21 (DE3) (Novagen, VWR International, Fontenay-sous-Bois, France) were used for cloning experiments and over-expression of GUA-1, respectively. *P. aeruginosa* KG2505 (20), an AmpC-deficient PAO1 mutant (*ampC*::ΩSm, *mexA::res*ΩSm), was used as the host for susceptibility testing.

### Antimicrobial agents, susceptibility testing, and microbiological techniques

Antimicrobial susceptibilities were determined by the disk diffusion technique on Mueller-Hinton agar (Bio-Rad, Marnes-La-Coquette, France) and by MIC determination, using E-tests (BioMérieux, Paris, France) and broth microdilution (Sigma–Aldrich, Saint-Quentin-Fallavier, France) as indicated in Table 1. Susceptibility results were interpreted according to EUCAST guidelines, updated in 2025 (http://www.eucast.org).

### PCR, cloning experiments, and DNA sequencing

The whole genome of *P. guariconensis-like* 65411, which was assembled using short- (Illumina, Evry, France) and long-read (MinION, Oxford Nanopore, UK) sequences using Unicycler (5) and available at Genbank nucleotide sequence database under the Bioproject PRJNA1150136 (accession number JBJGXE000000000), was used to design the primers. Total DNA of *P. guariconensis* 65411 clinical isolate was extracted using the QIAamp DNA mini kit (Qiagen, Courtaboeuf, France) from overnight cultures. The *bla*_GUA-1_ gene, which corresponds to the coordinates 1,499,205–1,498,330 of the genome of *P. guariconensis*-like 65411 was PCR amplified using primers Pre-GUA-1-F and GUA-1-R (Table S1) and cloned into the pCR-Blunt II-TOPO plasmid (Invitrogen, Illkirch, France), downstream of the pLac promoter, in the same orientation, resulting in pTOPO-GUA-1. The recombinant pTOPO-GUA-1 plasmid was electroporated into *E. coli* TOP10 cells, and electroporants were selected on TSA plates containing kanamycin (50 μg/mL).

The *bla*_GUA-1_ gene was PCR amplified using primers pre-GUA-1-BamH1 and GUA-1-XbaI (Table S1) and cloned into the shuttle broad-host-range vector pUCP24 with the NEBuilder HiFi DNA Assembly Master kit (New England BioLabs, Evry), resulting in plasmid pUCP24-GUA-1 and subsequently electroporated into *E. coli* TOP10 and *P. aeruginosa* KG2505 strains. Transformants were selected on Mueller-Hinton agar supplemented with gentamicin at 5 mg/L and 50 mg/L for *E. coli* and *P. aeruginosa* strains, respectively.

The *bla*_GUA-1_ gene fragment corresponding to the mature β-lactamase (from AA 28–291), as predicted by SignalP-5.0 (https://services.healthtech.dtu.dk/services/SignalP-5.0/), was cloned into the expression vector pET41b (+) (Novagen, VWR International, Fontenay-sous-Bois, France) using the PCR-generated fragment with primers NdeI-GUA-1_28–291_ and GUA-1-Δ-stop-XhoI (Table S1) and the NEBuilder HiFi DNA Assembly Cloning Kit (New England BioLabs Inc, Ozyme, Les Ulis, France), following the manufacturer’s instructions. The recombinant plasmid pET41-GUA-1 containing the mature GUA-1 β-lactamase added by a stretch of eight histidines at the C-terminus was transformed into the chemically competent *E. coli* strain BL21 (DE3) (Novagen), as previously described (21).

### β-Lactamase purification and steady-state kinetic parameters

An overnight culture of *E. coli* strain BL21 (DE3) harboring pET41b-GUA-1_28–291_ was used to inoculate 2 L of LB broth containing 50 µg/mL kanamycin. The bacteria were cultured at 37°C until reaching an OD of 0.6 at 600 nm. Expression of GUA-1 was induced overnight at 22°C with 0.2 mM IPTG, as previously described (22). GUA-1 was purified in two steps: a pseudo-affinity chromatography using an NTA-Ni column (GE Healthcare, Les Ulis, France) followed by a gel filtration step with 100 mM sodium phosphate buffer, pH 7, and 150 mM NaCl with a Superdex 75 column (GE Healthcare) (22). Protein purity was estimated by SDS-PAGE; pure fractions were pooled and dialyzed against 100 mM sodium phosphate buffer (pH 7.0) and concentrated using Vivaspin columns (GE Healthcare, Freiburg, Germany) until a concentration of 0.1 mg/mL. The protein concentrations were determined by measuring the OD at 280 nm and using the extinction coefficients obtained from the ProtParam tool (https://web.expasy.org/protparam/).

Kinetic parameters of purified GUA-1 were determined at 24°C in 100 mM sodium phosphate buffer (pH 7.0). The *k*_cat_ and *K*_m_ values were determined by analyzing hydrolysis of β-lactams under initial-rate conditions with an ULTROSPEC 2000 model UV spectrophotometer (GE Healthcare) using the Eadie–Hoffstee linearization of the Michaelis–Menten equation, as previously described (23). The β-lactams were purchased from Sigma-Aldrich (Saint-Quentin-Fallavier, France).

Various concentrations of clavulanic acid, tazobactam, and avibactam were preincubated with the enzyme for 3 min at 24°C before testing the rate of cephalothin (100 mM) hydrolysis. The 50% inhibitory concentrations (IC_50_) of these inhibitors were determined as the concentration of these inhibitors that inhibited hydrolytic activity by 50%.

### Mapping of the *bla*_GUA-1_ TSSs

Reverse transcription and rapid amplification of cDNA ends (5′ RACE; Invitrogen) were performed as previously described (24, 25). Five micrograms of total RNAs extracted using the Qiagen RNeasy Maxi kit (Qiagen, Les Ulis, France) from *P. guariconensis*-like 65411 were used for 5′ RACE experiments. Each 5′ RACE experiment was done in duplicate. Primers are detailed in Table S1. Single amplification products were cloned into pCR-Blunt II-TOPO plasmid (Invitrogen), as previously described, and inserts were sequenced from three independent colonies. The transcription initiation site (+1) was determined as the first nucleotide following the sequence of the adapter primer.

### RT-PCR experiments

To quantify transcription of the *bla*_GUA-1_ gene in *P. guariconensis*-like 65411, a one-step quantitative RT-PCR kit (QuantiFast SYBR-Green, Qiagen) was used as previously described (24, 25). DNA traces were removed by treating RNA with DNase I during the extraction protocol as recommended (Qiagen). The quantity of amplified product was monitored by the detection of fluorescence energy emitted at 530 nm by SYBR-Green. The threshold cycle number (Ct) was defined as the number of PCR cycles at which the fluorescence generated by the binding of SYBR-Green dye to double-stranded DNA begins to increase exponentially. The melting curve for each RT-PCR product was analyzed to determine the specificity of the amplified products. Each experiment was done in triplicate, and each quantification was done in triplicate. Transcriptional levels were standardized relative to the transcriptional level of the constitutive *Na+/H* + antiporter gene (Fig. 4; Table S1). The transcriptional levels determined by quantitative RT-PCR of the *bla*_GUA-1_ gene were compared to those of the other β-lactamase genes present in *P. guariconensis*-like 65411 and the *Na+/H* + gene located upstream of the *bla*_GUA-1_ gene, and interpreted using 2^−ΔΔCT^ as previously described (24, 25).

### DNA sequencing, DNA, and protein analyses

PCR products were purified using Qiaquick PCR purification spin columns (Qiagen), and the inserts of the recombinant plasmids were sequenced on both strands on an ABI 3100 automated sequencer (Applied Biosystems). The nucleotide and the deduced protein sequences were analyzed with software available over the internet at the National Center of Biotechnology Information website (http://www.ncbi.nlm.nih.gov). Multiple protein sequence alignments and phylogenetic trees were carried out online by using the program Clustal W (https://www.genome.jp/tools-bin/clustalw) and PhyML v20160115 (11). Online tool SAPPHIRE (SAPPHIRE.CNN.pseudomonas; https://sapphire.biw.kuleuven.be/index.php) was used to predict potential promoter sequences upstream of the *bla*_GUA-1_ gene.

### Molecular modeling

Three-dimensional structures of GUA-1 and LUT-1 in apo form and in complex with cefotaxime and ceftazidime were generated with Protenix (26). In all predictions, the β-lactam ring is situated as expected in front of Ser70, in a position compatible with the nucleophilic attack. The molecular modeling analysis images were generated using UCSF Chimera X (27). Throughout the manuscript, the residue numbering followed the ABL numbering scheme introduced by Ambler et al. (28).

## SUPPLEMENTAL MATERIAL

Supplemental material aac.00126-26-s0001.docx Tables S1 and S2; Fig. S1 and S2.

ASM does not own the copyrights to Supplemental Material that may be linked to, or accessed through, an article. The authors have granted ASM a non-exclusive, world-wide license to publish the Supplemental Material files. Please contact the corresponding author directly for reuse.
