# Supplementary material for: Characterization of GUA-1, a chromosomally encoded ESBL from Pseudomonas guariconensis-like
Source: Antimicrob Agents Chemother. 2026 Jun 30;70(8):e00126-26. doi: 10.1128/aac.00126-26 (PMC13436361; doi:10.1128/aac.00126-26)
Supplement: Supplementary file 1 [file aac.00126-26-s0001.docx]

**Characterization of GUA-1, a chromosomally-encoded ESBL from Pseudomonas guariconensis-like**

**Saoussen OUESLATI,^a,b^ Reva NERMONT,^a,b^ Nadia JAIDANE,^c,d^ Delphine GIRLICH,^a^ Nahed AL LAHAM,^a,e^ Lamia TILOUCHE,^c,d^ Wejdene MANSOUR,^c,^ Marisa HAENNI,^f^ Bogdan I. IORGA**,^g,h^ **and Thierry NAAS^a,b,h,i,j^**

Supplemental material

Tables:2

Figures: 2

TABLE S1. Sequences of primers used in this study

| Primers | Sequence (5’→ 3’) | Usage | Melting temperature (°C) | GC content  (%) | Amplicon size  (bp) |
| --- | --- | --- | --- | --- | --- |
| Pre-GUA-1-F | TCTTCTCCAGGGAATACACC | Cloning into pTOPO | 60 | 50 | 896 |
| GUA-1 R | TCACACCTTCCAGGCCTC |  | 56 | 56 |  |
| Pre- GUA-1 -BamH1 | GATTCCCTGGAGAAGA**GGATCC**CCGGGTACC | Cloning into pUCP24 | 46 | 50 | 922 |
| GUA-1 -Xba1 | CCTGGAAGGTGTGA**TCTAGA**GTCGACCT |  | 42 | 57 |  |
| NdeI-GUA-1-28-291 | AAGGAGATATA**CATATG**TCCGACCCGGTTCACCAGC | Cloning into pEt41b | 62 | 52 | 825 |
| GUA-1-D-stop-XhoI | GGTGGTGGTG**CTCGAG**CACCTTCCAGGCCTCCACG |  | 64 | 57 |  |
| M13 Reverse | CAGGAAACAGCTATGA | Universal sequencing primers | 54 | 45 | 1116 |
| T7 | TAATACGACTCACTATAGGG |  | 60 | 50 |  |
| GUA-1-RT-F | ACATTCAGTCGCCGCCAACT | RT_PCR *bla*_GUA-1_ | 60.5 | 55.0 | 209 |
| GUA-1-RT-R | AAGGTACTGCACAGTGCGGC |  | 62.5 | 60.0 |  |
| DHA-1-RT-F | ATCGTTATGCTGCGCTCTGC | RT_PCR *bla*_DHA-1_ | 60.0 | 55.0 | 211 |
| DHA-1-RT-R | GACTGGGTGGTTATTGGCGA |  | 60.5 | 55.0 |  |
| CMY-16-RT-F | ACTGATTTCCGCTCTGCTGG | RT_PCR *bla*_CMY-16_ | 60.5 | 55.0 | 207 |
| CMY-16-RT-R | GTATTTTCAGTGACCGGCTG |  | 58.4 | 50.0 |  |
| OXA-1-RT-F | TATCTACAGCAGCGCCAGTG | RT_PCR *bla*_OXA-1_ | 60.5 | 55.0 | 262 |
| OXA-1-RT-R | CCAGATCTCCATTCCTTTGGG |  | 61.3 | 52.4 |  |
| OXA-10-RT-F | GCCGCATATGTAATTATCGCG | RT_PCR *bla*_OXA-10_ | 59.4 | 47.6 | 223 |
| OXA-10-RT-R | CTAGGCCGATAATTGCGTTG |  | 58.4 | 50.0 |  |
| OXA-204-RT-F | GTATTAGCCTTATCGGCTGTG | RT_PCR *bla*_OXA-204_ | 59.4 | 47.6 | 189 |
| OXA-204-RT-R | TGCTTGGTTCGCCCGTTTAA |  | 58.4 | 50.0 |  |
| NA/H-RT-F | CTTTCGCCAACTTCGAGCGT | RT_PCR *Na+/H+* | 60.5 | 55.0 | 198 |
| NA/H-RT-R | GGCCATCGTTGATCCAGAAA |  | 58.4 | 50.0 |  |
| GSP1- GUA | TGTCGCTGTATTGCAGTGCC | 5’RACE *bla*_GUA-1_ | 60.5 | 55.0 | 380-bp from ATG |
| GSP2- GUA | CATGCTTTTCAGTGACAGGC |  | 58.4 | 50.0 | 317-bp from ATG |
| GSP3- GUA | TTCGCTGCGATAAGACAACC |  | 58,4 | 50.0 | 87-bp from ATG |
| GSP1-Na | TAGATCAATGCGGGCACGGC | 5’RACE *Na+/H+* | 64.0 | 60.0 | 397-bp from ATG |
| GSP2-Na | CAAGCTGGAAAGCGCGCCTT |  | 64.0 | 60.0 | 339-bp from ATG |
| GSP3-Na | TCTCCATATCGAAGCCGTCA |  | 58.4 | 50.0 | 191-bp from ATG |

Table S2 : *Bla*_GUA-1_ containing *Pseudomonas* sp. genomes in Genbank databases

| **Isolates #** | **Origin** | **Country,**  **city** | **Collection Date** | **Genbank**  **Submission Date** | **BioProject** | **Level**  **(Size in bp)** | **Position of *bla*_GUA-1_ in sequence** | **Accession number** | **BioSample** | **Taxonomy**  **(as per authors)** | **Reference** |
| --- | --- | --- | --- | --- | --- | --- | --- | --- | --- | --- | --- |
| 65411 | Blood,  Human | Tunisia,  Sousse | 14/10/223 | 28/11/2024 | PRJNA1150136 | Chromosome  (5,873,621) | 1499205-1498330 | JBJGXE000000000 | SAMN43272032 | *Pseudomonas sp.* | Jaidane et al. (2025) |
| HLMP | Vermicompost | India,  Mandsaur | 03/2021 | 30/12/2024 | [PRJNA1111369](https://www.ncbi.nlm.nih.gov/bioproject/1111369) | Contig 1  (5,160,861) | 2831387-2832262 | [JBDLLR000000000](https://www.ncbi.nlm.nih.gov/nuccore/JBDLLR000000000) | [SAMN41389144](https://www.ncbi.nlm.nih.gov/biosample/SAMN41389144) | [*Pseudomonas sp.*](https://www.ncbi.nlm.nih.gov/taxonomy/3153767) | Unpublished |
| Marseille-P8916 | Osteoarticular,  Human | France,  Marseille | 28/09/21 | 01/10/2021 | PRJEB46721 | Contig 8 (210,116) | 67099-66224 | [CAKAKK000000000](https://www.ncbi.nlm.nih.gov/nuccore/CAKAKK000000000) | [SAMEA9543671](https://www.ncbi.nlm.nih.gov/biosample/SAMEA9543671) | [*Pseudomonas sp.*](https://www.ncbi.nlm.nih.gov/taxonomy/2866589) | Unpublished |
| S311-3 | *Gallus gallus,*  animal | Czech Republic,  Moravia | 2020 | 17/06/2022 | PRJNA844552 | Contig 4 (148,081) | 75194-76069 | [JAMYBI000000000](https://www.ncbi.nlm.nih.gov/nuccore/JAMYBI000000000) | SAMN29162773 | [*P. guariconensis*](https://www.ncbi.nlm.nih.gov/taxonomy/1288410) | Unpublished |
| S311-4 | *Gallus gallus,*  animal | Czech Republic,  Moravia | 2020 | 17/06/2022 | PRJNA844552 | Contig 6 (133,632) | 75211-76086 | [JAMYBJ000000000](https://www.ncbi.nlm.nih.gov/nuccore/JAMYBJ000000000) | SAMN29162774 | [*P. guariconensis*](https://www.ncbi.nlm.nih.gov/taxonomy/1288410) | Unpublished |
| S311-5 | *Gallus gallus,*  animal | Czech Republic,  Moravia | 2020 | 17/06/2022 | PRJNA844552 | Contig 7 (133,632) | 75211-76086 | [AMYBR000000000](https://www.ncbi.nlm.nih.gov/nuccore/JAMYBR000000000) | SAMN29162782 | [*P. mosselii*](https://www.ncbi.nlm.nih.gov/taxonomy/78327) | Unpublished |
| S311-8 | *Gallus gallus,*  animal | Czech Republic,  Moravia | 2020 | 17/06/2022 | PRJNA844552 | Contig 15 (103,058) | 30153-31028 | [JAMYBZ000000000](https://www.ncbi.nlm.nih.gov/nuccore/JAMYBZ000000000) | SAMN29162790 | [*P. putida*](https://www.ncbi.nlm.nih.gov/taxonomy/303) | Unpublished |
| S332-4 | *Gallus gallus,*  animal | Czech Republic,  Moravia | 2020 | 17/06/2022 | PRJNA844552 | Contig 12  (148039) | 71851-72726 | [JAMYBH000000000](https://www.ncbi.nlm.nih.gov/nuccore/JAMYBH000000000) | SAMN29162772 | [*P. guariconensis*](https://www.ncbi.nlm.nih.gov/taxonomy/1288410) | Unpublished |
| S4446-10 | *Gallus gallus,*  animal | Czech Republic,  Moravia | 2020 | 17/06/2022 | PRJNA844552 | Contig 10 (144,892) | 69668-68793 | [JAMYBK000000000](https://www.ncbi.nlm.nih.gov/nuccore/JAMYBK000000000) | SAMN29162775 | [*P. guariconensis*](https://www.ncbi.nlm.nih.gov/taxonomy/1288410) | Unpublished |

*bla*_GUA-1.1__65411 ATGAAGCCGACATTCAGTCGCCGCCAACTGATCCAGGGCGCCATGGGGGGCTCGGTCATGCTGGCAACGCCCGCCTGGGC 80

*bla*_GUA-1.1__HLMP --------------------------------------------------------------------------------

*bla*_GUA-1.2__P8916 --------------------------------------------------------------------------------

*bla*_GUA-1.2__S311_3 --------------------------------------------------------------------------------

*bla*_GUA-1.2__S311-4 --------------------------------------------------------------------------------

*bla*_GUA-1.2__S311-5 --------------------------------------------------------------------------------

*bla*_GUA-1.2__S311-8 --------------------------------------------------------------------------------

*bla*_GUA-1.2__S332-4 --------------------------------------------------------------------------------

*bla*_GUA-2.0__S4446-10 --------------------------------------------------------------------------------

*bla*_GUA-1.1__65411 GTCCGACCCGGTTCACCAGCGCTTGCAGCACCTGGAGCAGGCGTTGGGCGGTACCCTGGGGCTTTGGGCGCTCGACACGG 160

*bla*_GUA-1.1__HLMP --------------------------------------------------------------------------------

*bla*_GUA-1.2__P8916 --------------------------------------------------------------------------------

*bla*_GUA-1.2__S311_3 --------------------------------------------------------------------------------

*bla*_GUA-1.2__S311-4 --------------------------------------------------------------------------------

*bla*_GUA-1.2__S311-5 --------------------------------------------------------------------------------

*bla*_GUA-1.2__S311-8 --------------------------------------------------------------------------------

*bla*_GUA-1.2__S332-4 --------------------------------------------------------------------------------

*bla*_GUA-2.0__S4446-10 --------------------------------------------------------------------------------

*bla*_GUA-1.1__65411 GAAGCGGCCGGCGGTTGTCTTATCGCAGCGAAGCGCGTGCCGCACTGTGCAGTACCTTCAAGGTCGTGCTGGCCGCTGCC 240

*bla*_GUA-1.1__HLMP --------------------------------------------------------------------------------

*bla*_GUA-1.2__P8916 --------------------------------------------------------------------------------

*bla*_GUA-1.2__S311_3 --------------------------------------------------------------------------------

*bla*_GUA-1.2__S311-4 --------------------------------------------------------------------------------

*bla*_GUA-1.2__S311-5 --------------------------------------------------------------------------------

*bla*_GUA-1.2__S311-8 --------------------------------------------------------------------------------

*bla*_GUA-1.2__S332-4 --------------------------------------------------------------------------------

*bla*_GUA-2.0__S4446-10 ------------A-------------------------------------------------------------------

*bla*_GUA-1.1__65411 ATCCTCCAGCAGGACCAGGCCGAGCCCGGCCTGCTGGAGCGCCGCATCGCCTACACCGCCGAGCAGTTGGTGGTCTATTC 320

*bla*_GUA-1.1__HLMP --------------------------------------------------------------------------------

*bla*_GUA-1.2__P8916 --------------------------------------------------------------------------------

*bla*_GUA-1.2__S311_3 --------------------------------------------------------------------------------

*bla*_GUA-1.2__S311-4 --------------------------------------------------------------------------------

*bla*_GUA-1.2__S311-5 --------------------------------------------------------------------------------

*bla*_GUA-1.2__S311-8 --------------------------------------------------------------------------------

*bla*_GUA-1.2__S332-4 --------------------------------------------------------------------------------

*bla*_GUA-2.0__S4446-10 --------------------------------------------------------------------------------

*bla*_GUA-1.1__65411 GCCTGTCACCGAAAAGCATGTCGCAACGGGCATGAGTGTCGCTGAGCTCTGTGCAGCGGCACTGCAGTACAGCGACAACA 400

*bla*_GUA-1.1__HLMP --------------------------------------------------------------------------------

*bla*_GUA-1.2__P8916 ---------T--------------------------------------------------------A-------------

*bla*_GUA-1.2__S311_3 ---------T--------------------------------------------------------A-------------

*bla*_GUA-1.2__S311-4 ---------T--------------------------------------------------------A-------------

*bla*_GUA-1.2__S311-5 ---------T--------------------------------------------------------A-------------

*bla*_GUA-1.2__S311-8 ---------T--------------------------------------------------------A-------------

*bla*_GUA-1.2__ S332-4 ---------T--------------------------------------------------------A-------------

*bla*_GUA-2.0__S4446-10 ------------------------------------------------------------------A-------------

*bla*_GUA-1.1__65411 CGGCGGCGAATCTCTTGCTGGGAGTGGTCGGTGGGCCGTCGAAGCTCACCGCCTATGCCCGCAGCATCGGCGACCCGGCC 480

*bla*_GUA-1.1__HLMP --------------------------------------------------------------------------------

*bla*_GUA-1.2__P8916 --------------------------------------------------------------------------------

*bla*_GUA-1.2__S311_3 --------------------------------------------------------------------------------

*bla*_GUA-1.2__S311-4 --------------------------------------------------------------------------------

*bla*_GUA-1.2__S311-5 --------------------------------------------------------------------------------

*bla*_GUA-1.2__S311-8 --------------------------------------------------------------------------------

*bla*_GUA-1.2__S332-4 --------------------------------------------------------------------------------

*bla*_GUA-2.0__S4446-10 --------------------------------------------------------------------------------

*bla*_GUA-1.1__65411 TTTCGCCTCGATCGATATGAACCGGCGTTGAACAGCGCGCTGCCTAATGACCCAAGGGATACCTCCACACCCTTGGCCAT 560

*bla*_GUA-1.1__HLMP --------------------------------------------------------------------------------

*bla*_GUA-1.2__P8916 --------------------------------------------C-----------------------------------

*bla*_GUA-1.2__S311_3 --------------------------------------------C-----------------------------------

*bla*_GUA-1.2__S311-4 --------------------------------------------C-----------------------------------

*bla*_GUA-1.2__S311-5 --------------------------------------------C-----------------------------------

*bla*_GUA-1.2__S311-8 --------------------------------------------C-----------------------------------

*bla*_GUA-1.2__S332-4 --------------------------------------------C-----------------------------------

*bla*_GUA-2.1__S4446-10 --------------------------------------------C-----------------------------------

*bla*_GUA-1.1__65411 GGGCGTCACCCTGCAGCGCCTGCTGTTGACGGATGGCCTGCCGGCGGCGGCCCGACTGCGCCTGCAGGACTGGCTCAAGG 640

*bla*_GUA-1.1__HLMP --------------------------------------------------------------------------------

*bla*_GUA-1.2__P8916 --------------------------------------------------------------------------------

*bla*_GUA-1.2__S311_3 --------------------------------------------------------------------------------

*bla*_GUA-1.2__S311-4 --------------------------------------------------------------------------------

*bla*_GUA-1.2__S311-5 --------------------------------------------------------------------------------

*bla*_GUA-1.2__S311-8 --------------------------------------------------------------------------------

*bla*_GUA-1.2__S332-4 --------------------------------------------------------------------------------

*bla*_GUA-2.0__S4446-10 --------------------------------------------------------------------------------

*bla*_GUA-1.1__65411 GCAATACCACAGGCGATACCCGTATCCGCGCCGGCTTGCCTGAGGGCTGGGTGGTTGGCGACAAGACCGGTTCGGGTGAC 720

*bla*_GUA-1.1__HLMP --------------------------------------------------------------------------------

*bla*_GUA-1.2__P8916 --------------------------------------------------------------------------------

*bla*_GUA-1.2__S311_3 --------------------------------------------------------------------------------

*bla*_GUA-1.2__S311-4 --------------------------------------------------------------------------------

*bla*_GUA-1.2__S311-5 --------------------------------------------------------------------------------

*bla*_GUA-1.2__S311-8 --------------------------------------------------------------------------------

*bla*_GUA-1.2__S332-4 --------------------------------------------------------------------------------

*bla*_GUA-2.0__S4446-10 --------------------------------------------------------------------------------

*bla*_GUA-1.1__65411 TACGGCGTTGCCAATGATGTCGGGATCATCTGGCCGCCAGGGCAGGCGCCCTGGATTCTGGCGATCTATACGCGAGGCCG 800

*bla*_GUA-1.1__HLMP --------------------------------------------------------------------------------

*bla*_GUA-1.2__P8916 --------------------------------------------------------------------------------

*bla*_GUA-1.2__S311_3 --------------------------------------------------------------------------------

*bla*_GUA-1.2__S311-4 --------------------------------------------------------------------------------

*bla*_GUA-1.2__S311-5 --------------------------------------------------------------------------------

*bla*_GUA-1.2__S311-8 --------------------------------------------------------------------------------

*bla*_GUA-2.0__S4446-10 --------------------------------------------------------------------------------

*bla*_GUA-1.1__65411 GGACAAGACTTCACCTTGGCGTAGCGACGTCATTGCCGAGGCGACCCGTGTGGTCGTGGAGGCCTGGAAGGTGTGA 876

*bla*_GUA-1.1__HLMP ----------------------------------------------------------------------------

*bla*_GUA-1.2__P8916 ----------------------------------------------------------------------------

*bla*_GUA-1.2__S311_3 ----------------------------------------------------------------------------

*bla*_GUA-1.2__S311-4 ----------------------------------------------------------------------------

*bla*_GUA-1.2__S311-5 ----------------------------------------------------------------------------

*bla*_GUA-1.2__S311-8 ----------------------------------------------------------------------------

*bla*_GUA-1.2__S332-4 ----------------------------------------------------------------------------

*bla*_GUA-2.0__S4446-10 ----------------------------------------------------------------------------

Figure S1: Nucleotide sequence alignment of *bla*_GUA-1_ gene from *P. guariconensis*-like 65411 from Tunisia with those of isolate HLMP from India, P8916 from France and S311_3, S311_4, S311_5, S311_8, S332-4 and S4446_10 from Czech Republic. Identical nucleotides are indicated by dashes. Start codon (ATG) and stop codon (TGA) are underlined.

57	70 73

. . .

```
GUA-1_65411    MKPTFSRRQLIQGAMGGSVMLATPAWASDPVHQRLQHLEQALGGTLGLWALDTGSGRRLSYRSEARAALCSTFKVVLAAA 80
GUA-1_HLMP     --------------------------------------------------------------------------------
GUA-1_P8916    --------------------------------------------------------------------------------
GUA-1_S311_3   --------------------------------------------------------------------------------
GUA-1_S311-4   --------------------------------------------------------------------------------
GUA-1_S311-5   --------------------------------------------------------------------------------
GUA-1_S311-8   --------------------------------------------------------------------------------
GUA-1_S332-4   --------------------------------------------------------------------------------
GUA-2_S4446-10 ---------------------------------------------------------Q----------------------

                                                               130 132
                                                                 . .
```

GUA-1_65411	ILQQDQAEPGLLERRIAYTAEQLVVYSPVTEKHVATGMSVAELCAAALQYSDNTAANLLLGVVGGPSKLTAYARSIGDPA 160

```
GUA-1_HLMP     --------------------------------------------------------------------------------
GUA-1_P8916    --------------------------------------------------------------------------------
GUA-1_S311_3   --------------------------------------------------------------------------------
GUA-1_S311-4   --------------------------------------------------------------------------------
GUA-1_S311-5   --------------------------------------------------------------------------------
GUA-1_S311-8   --------------------------------------------------------------------------------
GUA-1_S332-4   --------------------------------------------------------------------------------
GUA-2_S4446-10 --------------------------------------------------------------------------------

              161                179                                                   234 236
```

. . . .

```
GUA-1_65411    FRLDRYEPALNSALPNDPRDTSTPLAMGVTLQRLLLTDGLPAAARLRLQDWLKGNTTGDTRIRAGLPEGWVVGDKTGSGD 180
GUA-1_HLMP     --------------------------------------------------------------------------------
GUA-1_P8916    --------------------------------------------------------------------------------
GUA-1_S311_3   --------------------------------------------------------------------------------
GUA-1_S311-4   --------------------------------------------------------------------------------
GUA-1_S311-5   --------------------------------------------------------------------------------
GUA-1_S311-8   --------------------------------------------------------------------------------
GUA-1_S332-4   --------------------------------------------------------------------------------
GUA-2_S4446-10 --------------------------------------------------------------------------------

GUA-1_65411    YGVANDVGIIWPPGQAPWILAIYTRGRDKTSPWRSDVIAEATRVVVEAWKV 291
GUA-1_HLMP     ---------------------------------------------------
GUA-1_P8916    ---------------------------------------------------
GUA-1_S311_3   ---------------------------------------------------
GUA-1_S311-4   ---------------------------------------------------
GUA-1_S311-5   ---------------------------------------------------
GUA-1_S311-8   ---------------------------------------------------
GUA-1_S332-4   ---------------------------------------------------
GUA-2_S4446-10 ---------------------------------------------------
```

Figure S2: Amino acid sequence alignment of GUA-1 from *P. guariconensis*-like 65411 from Tunisia with those of isolate HLMP from India, P8916 from France and S311_3, S311_4, S311_5, S311_8, and S4446_10 from Czech Republic. Identical amino-acids are indicated by dashes. Conserved motives among class A beta-lactamases are boxed. The R57Q AA change in GUA-2 is also indicated.

References

Jaidane N, Mansour W, Tilouche L, Châtre P, François P, Lupo A, Oueslati S, Jacquemin A, Lazzem A, Karaborni S, Du Fraysseix L, Ben Lamine Y, Mahdhi N, Trabelsi A, Madec JY, Haenni M, Naas T. 2025. OXA-204 Carbapenemase in Clinical Isolate of Pseudomonas guariconensis, Tunisia. Emerg Infect Dis. 31:1197-1201.
